# Smart hydrogels for overcoming cancer multidrug resistance

**DOI:** 10.1186/s12943-026-02660-3

**Published:** 2026-04-11

**Authors:** Yong Wang, Baoyan Liu, Zou-Fang Huang, Harsh Patel, Jinming Yu, Man Hu, Zhe-Sheng Chen

**Affiliations:** 1 Department of Pharmaceutical Sciences, College of Pharmacy and Health Sciences, St. John’s University, New York, 11439 USA; 2 Department of Radiation Oncology, Shandong Cancer Hospital and Institute, Shandong First Medical University and Shandong Academy of Medical Sciences, Jinan, Shandong 250117 China; 3 Department of Occupational Pulmonology, Shandong Academy of Occupational Health and Occupational Medicine, Shandong First Medical University Affiliated Occupational Disease Hospital (Shandong Occupational Disease Hospital), Jinan, Shandong 250062 China; 4 Department of Hematology, The Second Affiliated Hospital of Shenzhen University, The People’s Hospital of Baoan, Shenzhen, Guangdong 518101 China

**Keywords:** Smart hydrogels, Multidrug resistance, Matrix normalization, Metabolic reprogramming, Immunotherapy

## Abstract

Multidrug resistance (MDR) remains the principal impediment to curative oncology, driven by complex interplays between cancer cells and the tumor microenvironment (TME). While nanomedicines have sought to overcome these delivery barriers, their clinical translation is often hampered by the heterogeneity of the enhanced permeability and retention (EPR) effect and by inefficient intratumoral delivery. In this review, we argue that overcoming MDR requires a transition beyond traditional passive drug delivery, advocating active, localized remodeling of the tumor ecosystem. Next-generation injectable hydrogels are increasingly recognized as localized viscoelastic niches that combine controlled intratumoral retention with the capacity to actively modulate biological responses within tumor TME. By converging principles of mechanobiology and immunometabolism, these hydrogels enable a multi-tiered strategy to dismantle multidimensional MDR. This approach begins with the biomechanical softening of the extracellular matrix to decouple mechanotransduction driven by Yes-associated protein (YAP) and transcriptional coactivator with PDZ-binding motif (TAZ), followed by the metabolic disruption of hypoxia-driven bioenergetics. Beyond the extracellular landscape, nanogel-enabled trafficking allows payloads to circumvent intracellular sequestration and efflux transporters, while immunomodulatory niches mobilize antitumor immunity through in situ vaccination and myeloid reprogramming. Finally, we evaluate the integration of artificial intelligence-driven design and patient-derived organoids as a technical bridge to reconcile laboratory ingenuity with clinical utility, aiming to transform the TME into a vulnerable therapeutic target.

**Supplementary Information:**

The online version contains supplementary material available at 10.1186/s12943-026-02660-3.

## Introduction

Despite the continued expansion of available anticancer therapies, multidrug resistance (MDR) remains a major barrier to durable treatment responses and curative outcomes, contributing substantially to cancer mortality, particularly in patients with therapy-resistant advanced malignancies [1, 2]. Historically, MDR has been largely explained by classical MDR mechanisms—specifically, cell-intrinsic processes dominated by the overexpression of ATP-binding cassette (ABC) efflux transporters such as P-glycoprotein (P-gp/ABCB1/MDR1) [3, 4]. However, advances in high-resolution multi-omics and spatial transcriptomics have expanded this view. MDR is now increasingly recognized as a complex resistance phenotype emerging from dynamic interactions between tumor cells and the heterogeneous tumor microenvironment (TME) [5]. Conceptually, this expanded paradigm can be organized along three complementary axes: (i) temporal origin, distinguishing intrinsic (pre-existing) from acquired resistance [6, 7]; (ii) mechanistic drivers, separating classical transporter-mediated efflux from non-transporter mechanisms such as EMT-associated stemness and metabolic rewiring [8–10]; and (iii) spatial context, contrasting local resistance governed by TME-derived physical and biochemical barriers with systemic resistance arising from suboptimal pharmacokinetics [11–13]. Consistent with this perspective, accumulating evidence indicates that the TME functions as a multifaceted protective barrier, with resistance mechanisms extending far beyond the cancer cell membrane [10, 13, 14]. At the biophysical level, aberrant extracellular matrix (ECM) stiffness and elevated solid stress not only hinder drug penetration but also activate mechanotransduction pathways that reinforce cellular survival [15, 16]. This structural barrier is compounded by a hostile metabolic landscape: hypoxic, lactate-rich niches fuel malignant bioenergetics while imposing a metabolic blockade on effector T cells [17, 18]. Concurrently, an immunosuppressive network—dominated by tumor-associated macrophages (TAMs) and myeloid-derived suppressor cells (MDSCs)—establishes an immunosuppressive niche that protects therapy-resistant cells from cytotoxic immune surveillance [19, 20]. Collectively, these interconnected barriers underscore that effectively overcoming MDR requires a strategic shift: moving beyond reductionist single-target strategies toward integrated interventions that dismantle the tumor-supportive ecosystem sustaining cancer cell survival.

Over the past decade, nanomedicine has been pursued as a means to overcome transport barriers in solid tumors, largely through reliance on the enhanced permeability and retention (EPR) effect [21–23]. Yet, the translational landscape has proven increasingly nuanced; while the EPR effect remains a foundational principle for passive targeting [24, 25], its clinical utility is often constrained by profound inter-patient and intra-tumoral heterogeneity [13, 26]. A seminal meta-analysis revealed that, on average, only 0.7% of administered nanoparticles infiltrates successfully into solid tumor parenchyma [27–29]. These findings reflect the inherent delivery inefficiencies of systemic administration rather than a categorical failure of the EPR effect itself. Conventional systemic carriers frequently lack the capacity to sustain therapeutic concentrations against elevated interstitial fluid pressure (IFP) or to actively reverse the hostile TME landscape [30, 31]. Beyond poor extravasation, systemic vectors face predatory sequestration by the mononuclear phagocytic system; recent evidence reveals that the liver can intercept up to 99% of administered particles, severely narrowing the therapeutic window and precipitating off-target toxicities [32]. These persistent bottlenecks necessitate a paradigm reimagining—shifting from passive, diffusion-governed systemic transport toward active locoregional modulation [33]. Within this evolving framework, injectable hydrogels offer distinct advantages beyond traditional nanomedicines. By functioning as a localized viscoelastic niche that mirrors host tissue mechanics, these scaffolds provide: (i) sustained, ultra-high local drug concentrations that are decoupled from systemic clearance [34, 35]; (ii) direct, immuno-interactive interfaces at the tumor-stroma boundary [36, 37]; and (iii) the capacity for spatiotemporal orchestration of bioactive cues to precisely remodel the TME from within [38–40].

**Fig. 1:**
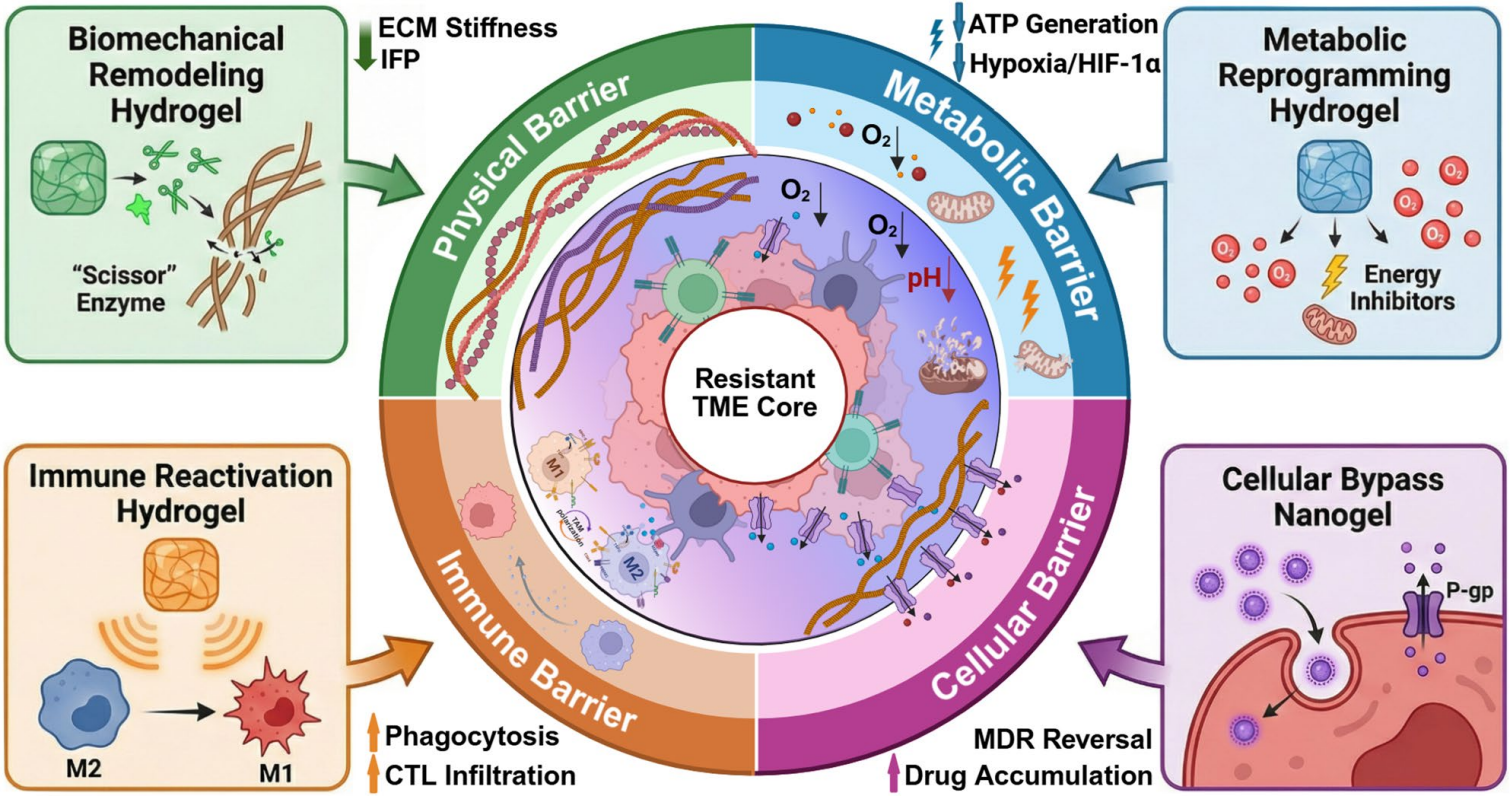
Programmable hydrogels as bio-responsive platforms for multifaceted tumor microenvironment. Four complementary mechanisms are illustrated. Biomechanically active hydrogels decrease extracellular matrix stiffness and interstitial fluid pressure, which improves tissue permeability and facilitates drug transport. In parallel, metabolically responsive hydrogels mitigate hypoxia and perturb tumor energy metabolism, weakening metabolically driven immune suppression. Nanogel-based intracellular delivery limits drug efflux and lysosomal trapping, allowing greater drug retention within drug resistant tumor cells. Immunoregulatory hydrogels locally bias macrophage polarization toward an antitumor phenotype and increase cytotoxic T-cell infiltration. Created with BioRender.com

Crucially, modern smart hydrogels have transcended their traditional roles as inert reservoirs, emerging instead as dynamic biological modulators. By sensing and orchestrating responses to microenvironmental cues—such as acidic pH, overexpressed enzymes, or reactive oxygen species (ROS)—these platforms initiate bio-responsive cascades that fundamentally reverse therapy resistance mechanisms [39–41]. This review posits that hydrogels should be conceptualized as platforms for local control and systemic conversion, rather than standalone MDR solutions. Effectively overcoming MDR requires a holistic strategy that sequentially dismantles the defensive hierarchy of the tumor ecosystem across four interconnected dimensions (systematically conceptualized in Fig. 1 and supported by the quantitative evidence map in Table 1). This process begins at the physical barrier, where dynamic matrix softening decouples mechanotransduction and restores hydraulic perfusion [42–44]. It then extends to the metabolic barrier, as in situ interventions disrupt the bioenergetic sanctuary by relieving hypoxia and nutrient scarcity [45–49]. At the subcellular level, this strategy circumvents efflux transporters and limits lysosomal sequestration through intelligent trafficking [50–53]. Beyond tumor cells, it engages the immune barrier to reinitiate the cancer-immunity cycle. While intratumoral depots primarily manage locoregional barriers, they act as biochemical anchors to prime systemic immunity or sensitize tumors to multi-modal therapeutic interventions, thereby addressing both primary lesions and potentially disseminated micro-metastases [54–57]. This review, guided by a semi-systematic literature selection process (covering 2013–2026) that prioritizes mechanistic and quantitative studies, evaluates the physicochemical principles governing the design of these intelligent platforms. We further provide a systematic framework for modulating the interconnected barriers of the TME, with the aim of rendering recalcitrant tumors more receptive to multi-modal therapeutic interventions.

**Table 1:** Quantitative evidence map linking TME barriers, hydrogel engineering strategies, and measurable therapeutic readouts

TME Dimension	TME Barrier & Mechanism	Hydrogel Engineering Strategy	Measurable Readouts (Quantitative)	Mechanistic Outcome	Evidence Level	Ref.
Physical Barrier	Matrix Stiffness: High stiffness (mimicking malignant breast tissue) activates YAP1/EMT to drive chemoresistance.	Magneto-Responsive Softening: Wireless, reversible stiffness switching via magnetic microparticles (GHAM hydrogel).	ΔStorage Modulus (G’): Switching between ~ 500 Pa (soft) and ~ 2700 Pa (stiff); ΔCell Density: ~30% reduction in cell density upon softening; Drug Sensitivity: Dead cell % increased from ~ 60% to ~ 80% following matrix softening.	Mechanical Rescue: Reversible downregulation of YAP1, TWIST1, and SNAI1/2; Reversal of hypoxia markers (HIF-1α, Nrf2) and EMT markers (Vimentin down, E-cadherin up).	3D MCF-7 Spheroids; Longitudinal single-population tracking; Correlation with TCGA & METABRIC patient databases.	[42]
	Solid Stress & High IFP: Excessive collagen in ECM leads to vessel compression and high IFP, blocking drug penetration.	Enzymatic Digestion: pH-triggered sequential release of collagenase and nanodrugs from polyelectrolyte hydrogel (CD@IPH).	Release Kinetics: ~50% collagenase released within 24 h at pH 6.5; Penetration Depth: Homogeneous distribution at 30–70 μm depth in 3D MCS vs. peripheral accumulation in controls.	ECM Remodeling: Targeted degradation of Type I Collagen; Reduction of mechanical barriers to restore deep nanoparticle infiltration as evidenced by Photoacoustic (PA) imaging.	3D Multicellular Spheroids (MCS); In vivo HepG2/4T1 tumor-bearing mice.	[44]
Metabolic Barrier	Hypoxic Sanctuary: Hypoxia stabilizes HIF-1α, which transcriptionally upregulates ABC transporters (e.g., P-gp/ABCB1), causing cellular drug efflux and resistance.	Self-Supplied Oxygenation: Acid-triggered hydrolysis of encapsulated CaO_2_ nanoparticles combined with Catalase (CAT).	↑ Oxygen Level: pO_2_ from ~ 2.1 mmHg to ~ 24.3 mmHg within 24 h; ↓ HIF-1α: ~65% reduction in protein expression.	HIF-1α Degradation: Reverses efflux pump expression (P-gp down ~ 50%); restores therapeutic sensitivity in recalcitrant cores.	3D Hypoxic Spheroids; In vivo 4T1 Orthotopic mouse; Photoacoustic (sO_2_) imaging.	[45, 46]
	Lactate Accumulation: Lactate export creates an acidic niche that inhibits effector T cell glycolysis and cytotoxic function.	Metabolic Scavenging: TME-triggered enzymatic consumption of lactate via encapsulated Lactate Oxidase (LOX) and Catalase (CAT).	↓ Lactate Concentration: From ~ 10.5 mM to ~ 2.8mM; ↑ Intratumoral pH: From 6.5 to ~ 7.1; ↑ T cell fitness: ~3.2-fold increase in CD8^+^ T cell infiltration.	Immune Metabolism Reset: Reversing the acidic blockade restores the bioenergetic fitness of effector T cells, re-initiating the cancer-immunity cycle.	3D Tumor Spheroids; Syngeneic 4T1 breast cancer mouse model; Flow cytometry for T-cell profiling.	[47–49]
Cellular Defense	Efflux Transporters: Overexpression of ABC transporters (e.g., P-gp/ABCB1) actively extrudes chemotherapeutics.	Micellar Bypass Strategy: TPGS/CS-CA-based ES-Cu nanoparticles designed to bypass efflux pumps.	Resistance Index (RI): 0.8–1.3 for ES-Cu NPs in multiple resistant lines (vs. >800 for paclitaxel); Docking Score: ES-Cu (-6.230) vs. Paclitaxel (-11.018), indicating low P-gp binding affinity.	Efflux Evasion: ES-Cu acts as a non-substrate for P-gp, maintaining therapeutic concentrations without compromising pump activity in healthy tissues.	DU145TXR, PC3TXR, A549TXR drug-resistant lines; In silico induced-fit molecular docking.	[50]
	Intracellular Sequestration: Endosomal entrapment and nuclear envelope prevent delivery to nucleus-localized lncRNAs.	Dual-Responsive Nuclear Delivery: pH-responsive PDPA-based NPs with NTPA peptide for endosomal escape and nuclear entry.	pH-Triggered Release: ~60% siRNA release at pH 6.0 within 12 h (vs. ~20% at pH 7.4); Nuclear Entry: >4-fold increase in nuclear siRNA fluorescence intensity from 4 h to 8 h.	Precision Trafficking: Enhanced escape via “proton sponge effect” followed by nucleus-specific silencing of lncMALAT1 (> 80% at 30nM).	Bel7402-DR cisplatin-resistant cells; In vivo xenograft tumor-bearing nude mice.	[51–53, 58]
Immune Barrier	“Cold” Tumor Phenotype: Lack of tumor antigens and dominant M2-TAMs prevent T-cell priming and infiltration.	iGEL In Situ Vaccination: NIR-triggered local release of cabazitaxel (ICD inducer) and TLR 7/8 agonist from ROS-responsive PVA-TSPBA gel.	↑ DC Maturation: 69.0 ± 3.1% (CD11c^+^CD80^+^CD86^+^); ↑ T-cell Infiltration: 40.5 ± 2.8% CD8^+^ T cells (of CD45^+^ cells); TAM M1-Polarization: ~26-fold increase in nos2 mRNA level.	Cycle Reignition: Triggers potent ICD to release neoantigens; synergistically activates TLR pathways to bridge innate and adaptive immunity; successfully reverses the immunosuppressive TME.	Syngeneic B16F10 & 4T1 models; B16F10-OVA for antigen-specific responses; Spontaneous metastatic models.	[54, 55]
	Innate Silencing: Suppression of the cGAS-STING pathway inhibits Type I interferon production, leading to immune evasion.	STING Activation Niche: Intratumoral self-assembly of nanotubes (CPT-iRGD) for the extended release of c-di-AMP (CDA).	Survival Rate: 100% survival (GL-261 model); ↑ CTL Recruitment: 23.9 ± 3.4% CD8^+^ T cells (vs. 14.7% for free drug); Retention: CDA detectable in tumor for 35 d.	Innate Awakening: Extended activation of STING/IFN-1 cascade transforms the TME from “cold” to “hot”; establishes long-term immunological memory and systemic surveillance.	Orthotopic GL-261 brain tumor; STING-deficient (Ting^gt/gt^) mice and cell depletion (NK/CD4/CD8) studies for causality.	[56, 57]

*ABC* ATP-binding cassette, *ABCB1* ATP-binding cassette sub-family B member 1, *CAT* Catalase, *c-di-AMP (CDA)* Cyclic di-adenosine monophosphate, *cGAS* Cyclic GMP-AMP synthase, *CTL* Cytotoxic T lymphocyte, *DC* Dendritic cell, *ECM* Extracellular matrix, *EMT* Epithelial-to-mesenchymal transition, *ES-Cu* Elesclomol-copper, *G’* Storage modulus, *HIF-1α* Hypoxia-inducible factor 1-alpha, *ICD* Immunogenic cell death, *IFP* Interstitial fluid pressure, *lncRNA* Long non-coding RNA, *LOX* Lactate oxidase, *MALAT1* Metastasis-associated lung adenocarcinoma transcript 1, *MCS* Multicellular spheroids, *NIR* Near-infrared, *NK* Natural killer, *NP* Nanoparticle, *PA* Photoacoustic, *P-gp* P-glycoprotein, *pO2* Partial pressure of oxygen, *RI* Resistance index, *STING* Stimulator of interferon genes, *TAM* Tumor-associated macrophage, *TLR* Toll-like receptor, *TPGS* D-α-tocopheryl polyethylene glycol 1000 succinate, *YAP1* Yes-associated protein 1

These hydrogel platforms are conceptualized as anchors for “local control and systemic conversion”; their efficacy against systemic micro-metastases depends on synergistic immune priming rather than as a standalone solution

## Breaching the physical barrier: biomechanical remodeling and matrix normalization

**Fig. 2:**
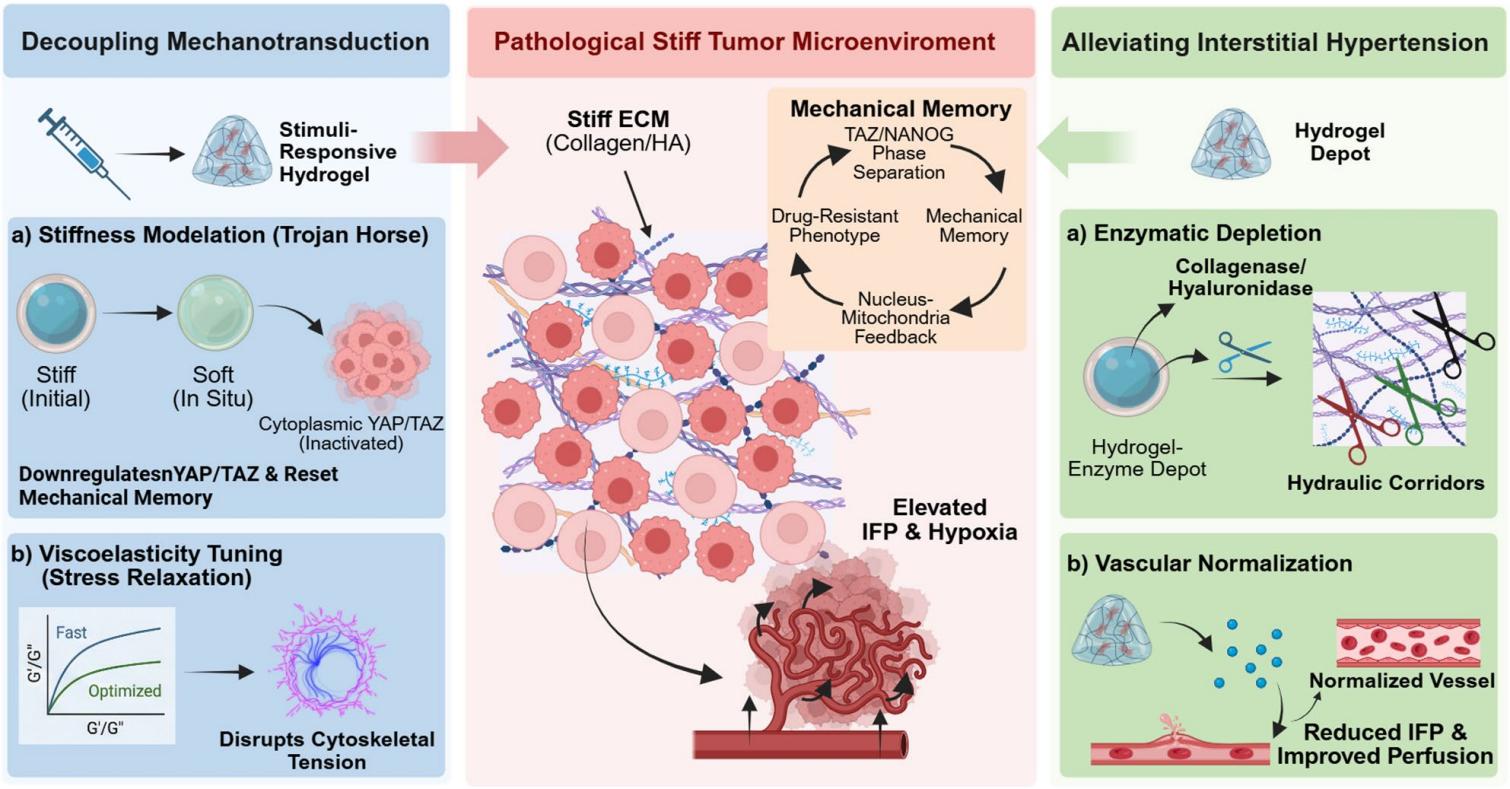
Strategic interventions for biophysical remodeling and matrix normalization. Overcoming the physical resistance of the tumor microenvironment (TME) requires a tactical shift toward addressing both cellular mechanotransduction and macroscopic fluid dynamics. One path focuses on uncoupling the cell from its rigid environment; by engineering hydrogels that soften on-demand or exhibit rapid stress relaxation, YAP/TAZ nuclear exclusion can be forced to achieve “mechanical rescue”. Alternatively, addressing the “pressure cooker” environment of the tumor core is essential. By utilizing localized enzymatic degradation to carve out hydraulic corridors, or employing vascular normalization agents, these platforms decompress solid stress and relieve interstitial fluid pressure (IFP). The mitigation of interstitial hypertension enables therapeutics and immune effectors to move beyond the tumor periphery and penetrate the interior core. Created with BioRender.com

The desmoplastic response within solid tumors yields a pathologically rigid ECM, a phenomenon now recognized not merely as a physical byproduct but as a fundamental driver of malignancy via Yes-associated protein (YAP) / transcriptional coactivator with PDZ-binding motif (TAZ)-dependent mechanotransduction [59–61]. Emerging evidence suggests this process is far more insidious, where transient stiffness exposure is hypothesized to establish long-lasting “mechanical memory” through physical states like TAZ/NANOG phase separation [62] and self-sustaining nucleus-mitochondria feedback loops that lock cells into drug-resistant phenotypes [63], although the causal hierarchy of these processes is still being clarified. Beyond its role as a structural barrier, the stiff TME actively confers multidrug resistance through complex crosstalk pathways, including activating fatty acid oxidation [64], ligand-independent epidermal growth factor receptor (EGFR) signaling [65], and stabilizing novel mechanosensors like ubiquitin-specific peptidase 9X (USP9X) [66]. These insights make it difficult to view hydrogels as mere passive supports. Once malignant phenotypes become mechanically entrenched, reversal likely depends on actively disrupting mechanotransduction (visualized conceptual flow in Fig. 2), for example through stimuli-responsive softening or materials with rapid stress relaxation that can relax cytoskeletal tension and weaken focal adhesion stability [67–69].

### Decoupling mechanotransduction: stiffness-tunable and stress-relaxing hydrogels

#### Dynamic stiffness modulation: the “Trojan horse” strategy

**Fig. 3:**
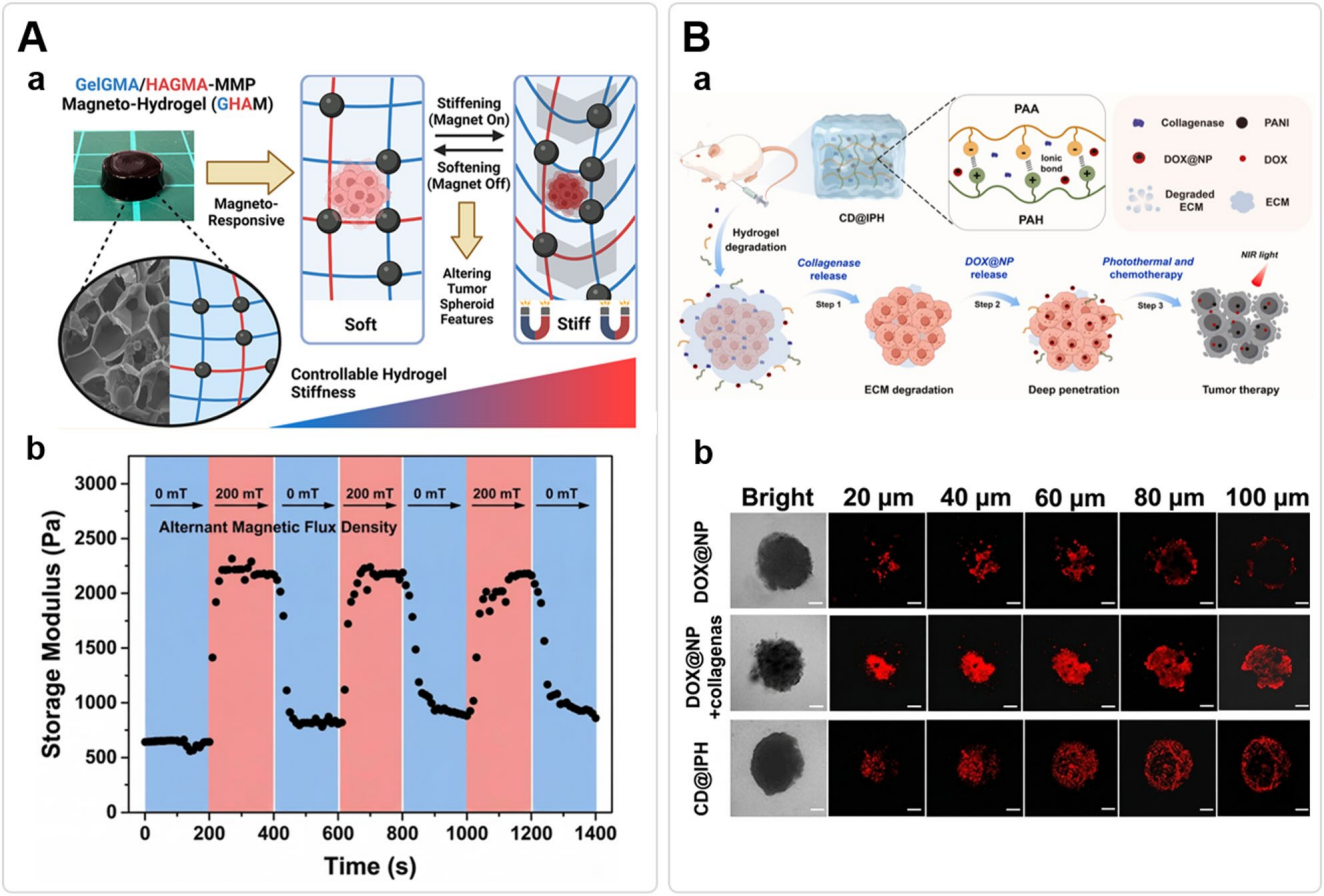
Hydrogel-mediated dynamic biomechanical remodeling and matrix normalization. **A** Dynamic stiffness modulation to modulate mechanotransduction. **a** Conceptual schematic of the magneto-responsive GHAM hydrogel, illustrating fabrication, morphology (photo/SEM inset), and the reversible stiffening mechanism to alter tumor spheroid features. **b** Rheological characterization, demonstrating rapid and repeatable switching of storage modulus between the soft (~ 560 Pa) and stiff (~ 2640 Pa) states under alternating magnetic flux density. (adapted from Ref [42]. ; Copyright 2023, American Chemical Society). **B** Enzymatic remodeling to reduce interstitial hypertension and enhance drug penetration. **a** Conceptual schematic of an injectable CD@IPH hydrogel delivering collagenase and DOX@NP, illustrating the multi-step process from matrix normalization (Step 1) to deep doxorubicin penetration (Step 2) and final therapy. **b** Representative in vitro Z-stack fluorescence images of tumor spheroids at varying depths (20–100 μm), demonstrating significantly enhanced intratumoral distribution of doxorubicin (DOX; red) with CD@IPH compared to DOX@NP alone. (adapted from Ref [44]. ; Copyright 2025, Elsevier)

Static hydrogels fail to recapitulate the spatiotemporal dynamics of tumor mechanics. To address this, a new generation of stimuli-responsive hydrogels has been engineered to perform an in situ “mechanical rescue.” These materials act as a “Trojan Horse,” initially maintaining high stiffness for retention and localization, before transitioning to a soft state that mimics healthy tissue to downregulate the tension-dependent YAP/TAZ axis [70]. The therapeutic relevance of such “softening” is fundamentally dictated by the magnitude of the mechanical transition. While healthy soft tissues (e.g., brain or adipose) typically reside in the Pascal (Pa) regime (0.1–1.0 kPa), malignant stiffening often escalates the TME into the kilo-Pascal (kPa) regime (> 10–100 kPa). Consequently, a biologically meaningful “rescue” requires hydrogels capable of bridging these distinct regimes—as precisely quantified by oscillatory rheology or atomic force microscopy (AFM)—to effectively deactivate cellular mechanosensors. For instance, Shou et al. developed a dynamic magneto-softening hydrogel (GHAM) whose stiffness could be wirelessly and reversibly tuned between ~ 560 Pa and ~ 2640 Pa (Fig. 3A), a range specifically chosen to mimic the transition from healthy to malignant breast tissues. They demonstrated that in situ matrix softening effectively reversed the malignant transformation of cancer cells, downregulated YAP1, and significantly potentiated drug efficacy by increasing the percentage of dead cells from approximately 60% to 80% following softening [42]. Similarly, leveraging the TME’s enzymatic repertoire, collagen-based hydrogels designed to undergo enzymatic softening have been shown to restore contact inhibition and enhance radiosensitivity in hepatocellular carcinoma cells [71].

More recently, these mechanobiological insights have been linked to measurable therapeutic effects in vivo. Wu et al. demonstrated that collagenase-driven matrix softening synergized with lenvatinib to inhibit tumor growth in vivo. Further analysis suggested that lowering matrix stiffness interfered with stiffness-associated mitochondrial fission and mitophagy, weakening cytoprotective programs and increasing tumor cell sensitivity to apoptosis [72]. Beyond its direct impact on tumor cells, mechanical modulation also shapes the immune compartment of the TME. For example, a near-infrared (NIR)-responsive hydrogel system was shown to dynamically modulate substrate stiffness to direct macrophage polarization, highlighting the potential of dynamic hydrogels to simultaneously dismantle the physical barrier and reverse immune suppression [73].

#### Beyond stiffness: viscoelasticity and stress relaxation as therapeutic targets

While matrix stiffness (G′) has long dominated studies of mechanotransduction, a series of recent studies have pointed to viscoelasticity—specifically the rate of stress relaxation—as an independent determinant of tumor behavior [74, 75]. This challenges the prevailing view that “softer is better,” as accumulating evidence shows that fast-relaxing matrices (mimicking the biophysical properties of certain brain and pancreatic tissues) actively drive malignancy. For instance, in glioblastoma (GBM), high-molecular-weight hyaluronic acid hydrogels with fast stress relaxation kinetics were shown to facilitate rapid invasion by enabling distinct leader–follower dynamics, a process driven by hyaluronidase-mediated matrix remodeling [76]. Similarly, in pancreatic ductal adenocarcinoma (PDAC), fast-relaxing hydrogels promoted an aggressive epithelial–mesenchymal transition (EMT) phenotype and proliferation. In vivo interference with this mechanosensing pathway led to a marked reduction in tumor burden, implicating stress relaxation sensors as druggable targets [77]. Furthermore, stress relaxation has also been linked to intrinsic ferroptosis resistance in colon cancer spheroids, creating a mechanically induced metabolic barrier that requires the use of specific nanozymes for resensitization [78].

Conversely, the tunable viscoelasticity of hydrogels provides a practical handle for therapeutic design, with implications for immune regulation and drug residence [36]. One influential study showed that matrix viscoelasticity can be framed as a “mechanical checkpoint” governing monocyte differentiation; unlike elastic matrices that drive pro-inflammatory polarization, viscoelastic matrices promote a restorative myeloid phenotype via the phosphoinositide 3-kinase gamma (PI3K-γ) pathway [79]. On this basis, recent designs such as cellulose nanocrystal–annealed hydrogels have tuned viscoelastic properties to improve the intratumoral retention of chemotherapeutics (doxorubicin) and metabolic inhibitors (2-deoxy-D-glucose), which was associated with improved control of melanoma recurrence [34]. Along the same lines, adjusting hydrogel viscoelasticity can bias stromal plasticity toward less desmoplastic states, providing another route toward stromal normalization [15]. Taken together, this points to a need for hydrogel systems that go beyond static mechanics and instead tune stress relaxation timescales to restrain mechanotransduction-driven invasion while supporting immune normalization.

### Alleviating elevated interstitial fluid pressure (IFP): matrix degradation and penetration enhancement

#### Enzymatic depletion: creating hydraulic corridors

Elevated IFP and solid stress, driven by dense collagen–hyaluronic acid (HA) networks, together give rise to a biophysical barrier that restricts therapeutic transport, largely by elevating hydraulic resistance [12, 80]. However, clinical efforts in systemic matrix depletion serve as a cautionary tale: the systemic hyaluronidase PEGPH20 (pegvorhyaluronidase alfa) failed to improve overall survival in Phase III trials for PDAC and raised significant safety concerns, underscoring that unselective stroma ablation can be clinically counterproductive [81]. To circumvent these risks, hydrogel platforms are now shifting toward localized remodeling, which generates “hydraulic corridors” through site-specific enzymatic depletion. One representative example is the “Jekyll and Hyde” nanogel–hydrogel hybrid, which remains inert in healthy tissue but becomes activated within the acidic, hyaluronidase-rich TME, enabling unidirectional release of tumor-penetrating cargo [82]. Pushing this concept further, recent studies describe a sequential “break-and-enter” strategy in which rapid collagenase release transiently disrupts the ECM, permitting the subsequent deep percolation of chemotherapeutic nanoparticles and resulting in improved combination efficacy (Fig. 3B) [44]. Importantly, the impact of such localized interventions extends beyond mere pharmacokinetics; dismantling the HA-rich niche using hydrogel-delivered hyaluronidase also suppresses invasive tumor phenotypes, effectively shifting the TME from a protective state toward one that is permeable and drug-responsive [83]. To ensure translational safety and avoid risks such as local hemorrhage, off-target matrix injury, or the unintended creation of metastasis-facilitating invasion corridors [81, 84], future hydrogel designs should prioritize matrix normalization rather than total ablation. This can be achieved through: (i) precise spatial confinement to the tumor site [55]; (ii) titratable release kinetics to prevent over-degradation; and (iii) integration with image-guided approaches to enable real-time monitoring of biomechanical responses [42].

#### Vascular normalization via gasotransmitters

While matrix depletion helps alleviate solid stress, the disorganized and leaky tumor vasculature maintains elevated IFP and hypoxia. Vascular normalization—strengthening vessel integrity to re-establish perfusion gradients—provides an unexpectedly effective way to lower IFP and improve drug delivery [85]. Intratumoral hydrogels delivering gasotransmitters, particularly nitric oxide (NO), are increasingly used for this purpose, provided that release kinetics are tightly regulated. At low concentrations, NO mimics physiological shear stress to upregulate VE-cadherin and recruit pericytes, whereas high concentrations exert cytotoxic effects [86, 87]. Yang et al. reported that a supramolecular NO depot releasing low-dose NO in a sustained manner normalized tumor vessels, increased pericyte coverage, and reduced vascular permeability, while higher-dose pulses acted as radiosensitizers [88]. Beyond reducing IFP, vascular normalization also reshapes the immune microenvironment. For example, an environmentally responsive hydrogel co-delivering an NO donor (RRx-001) and a stimulator of interferon genes (STING) agonist (c-di-AMP) enhanced CD8⁺ T-cell infiltration and relieved immunosuppression in tumors [56]. By shifting vasculature from a “leaky” to a “normalized” state, these hydrogels convert the tumor’s inefficient transport network into a more functional conduit for oxygen, drugs, and immune effectors.

## Remodeling the metabolic barrier: bioenergetic deprivation and hypoxia relief

**Fig. 4:**
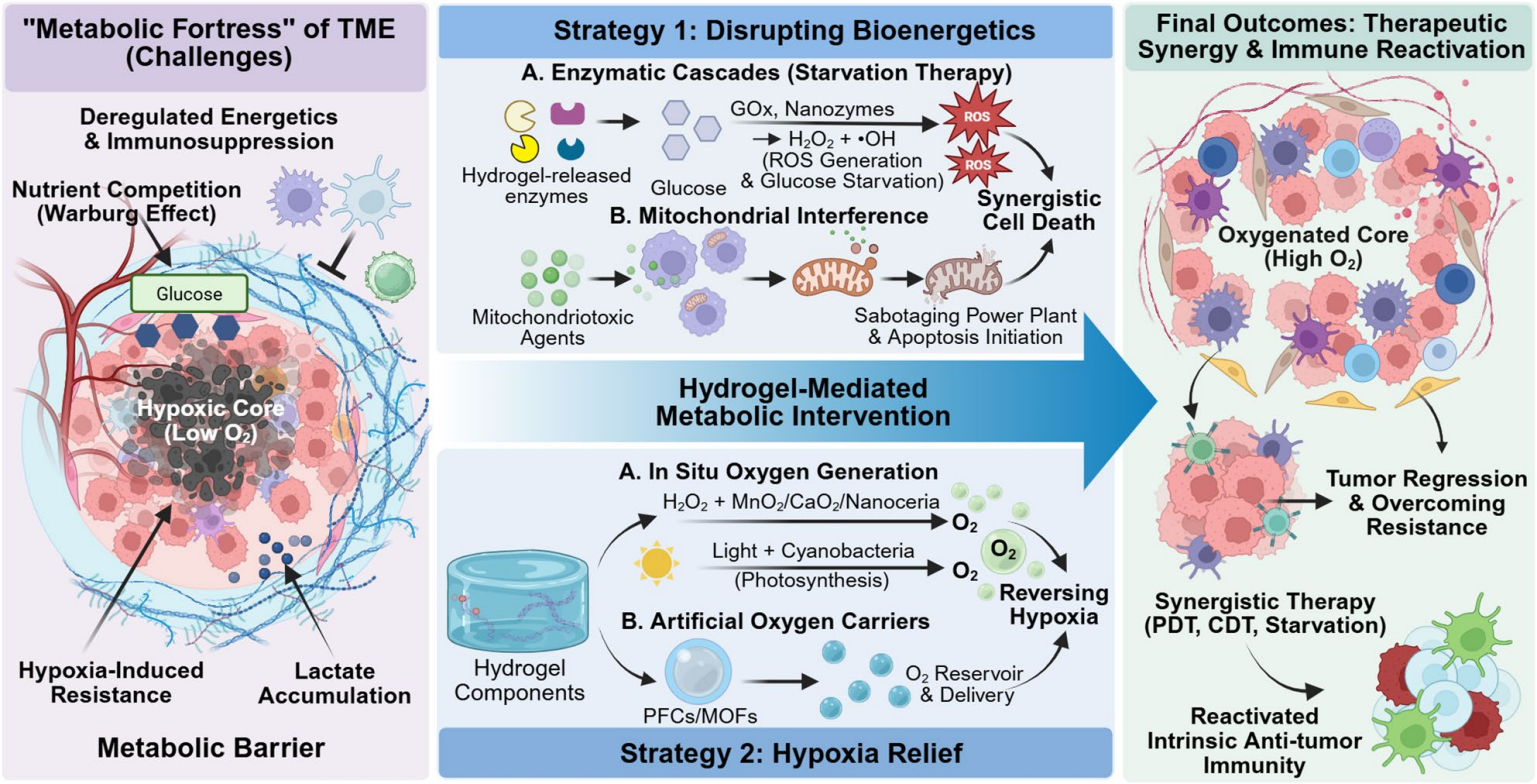
Hydrogel-enabled metabolic interventions for tumor microenvironment remodeling and immune reactivation. The schema highlights two linked approaches. Strategy 1 targets bioenergetic adaptability by inducing localized metabolic stress through enzymatic starvation (glucose oxidase) or direct mitochondrial ablation. Strategy 2 addresses hypoxia-driven resistance by supplying oxygen through in situ generation (photosynthetic cyanobacteria, MnO₂-based systems) or perfluorocarbon oxygen carriers. Together, these interventions reshape the metabolic landscape in ways that favor immune recovery. Created with BioRender.com

Beyond the structural normalization of the physical matrix, the subsequent challenge lies in addressing the metabolic barrier that persists within these remodeled boundaries. The metabolic landscape of the TME has evolved from a simple hypoxic niche into a multilayered metabolic constraint shaped by deregulated energetics [9]. This landscape is characterized by profound metabolic heterogeneity; regional gradients of oxygen and nutrients, often dictated by vessel proximity and varying diffusion limits, create micro-niches that demand precisely tailored interventions rather than broad, non-specific approaches. While the Warburg effect remains a hallmark of cancer, recent studies indicate that metabolic reprogramming extends beyond glycolysis to aberrant lipid and amino acid metabolism, which can contribute to therapeutic resistance [18, 89]. Crucially, the accumulation of oncometabolites such as lactate has been shown to act not merely as waste, but as a signaling cue that induces histone lactylation, an epigenetic modification that has been reported in preclinical models to potentially reinforce immune exhaustion and immunosuppression [90, 91]. This metabolic barrier thus may impose a dual challenge: nutrient competition that potentially limits anti-tumor effector function, and an acidic milieu that may favor resistant cancer cell populations [92]. Against this backdrop, advanced hydrogel systems are being explored as local tools for local control and systemic conversion in metabolic intervention. These scaffolds have been shown in preclinical studies to impose bioenergetic stress by restricting key substrates and perturbing mitochondrial function, while also potentially reshaping hypoxia and acidosis to support antitumor immunity (Fig. 4).

### Disrupting bioenergetics: mitochondrial targeting and starvation strategies

#### Starvation therapy via enzymatic cascades

While early strategies relied on simple encapsulation of glucose oxidase (GOx) to consume intratumoral glucose [93], recent work has shifted toward enzyme-assembled cascade systems that amplify metabolic stress. A representative example is the DNA adjuvant hydrogel reported by Zhao et al., which utilizes DNA double helices to precisely regulate the spatial distance between GOx and ferrocene catalysts to optimize the enzymatic cascade. In preclinical investigations, this spatial organization was reported to increase reactive oxygen species (ROS) generation by nine-fold, coupling glucose depletion with chemodynamic therapy (CDT) to trigger immunogenic cell death [94]. Multi-enzyme hydrogels provide a related route. In the GH@LDO platform incorporating CoMnFe layered double oxide nanozymes and GOx, it has been demonstrated in vivo that GOx-driven glucose oxidation starves tumor cells while producing hydrogen peroxide (H_2_O_2_) and gluconic acid. The generated H_2_O_2_ then feeds the nanozyme reaction to yield hydroxyl radicals and oxygen. Functionally, this cascade can contribute to the simultaneous depletion of bioenergy and alleviation of hypoxia, supporting a combined starvation–CDT–photothermal effect as observed in preclinical studies [95]. However, starvation therapy involves significant mechanistic trade-offs that warrant careful consideration. The enzymatic oxidation of glucose via GOx inevitably generates gluconic acid and H_2_O_2_, which, if not synchronized with adequate buffering or secondary scavenging, can potentially exacerbate local acidosis and oxidative stress [96]. In line with the acid-mediated invasion hypothesis, such intervention-induced heterogeneity in the TME may inadvertently select for acid-tolerant clones [97]. Moreover, emerging evidence suggests that glucose deprivation can trigger compensatory metabolic rewiring—such as a shift toward fatty acid oxidation [98] or glutaminolysis [99]—potentially driving tumor progression rather than regression [100]. Therefore, the integration of buffering components (e.g., pH-responsive scaffolds or inorganic acid-neutralizers) within the hydrogel depot is suggested to be essential to maintain a stable metabolic window and prevent the emergence of treatment-resistant phenotypes [101].

#### Mitochondrial interference and apoptosis initiation

**Fig. 5:**
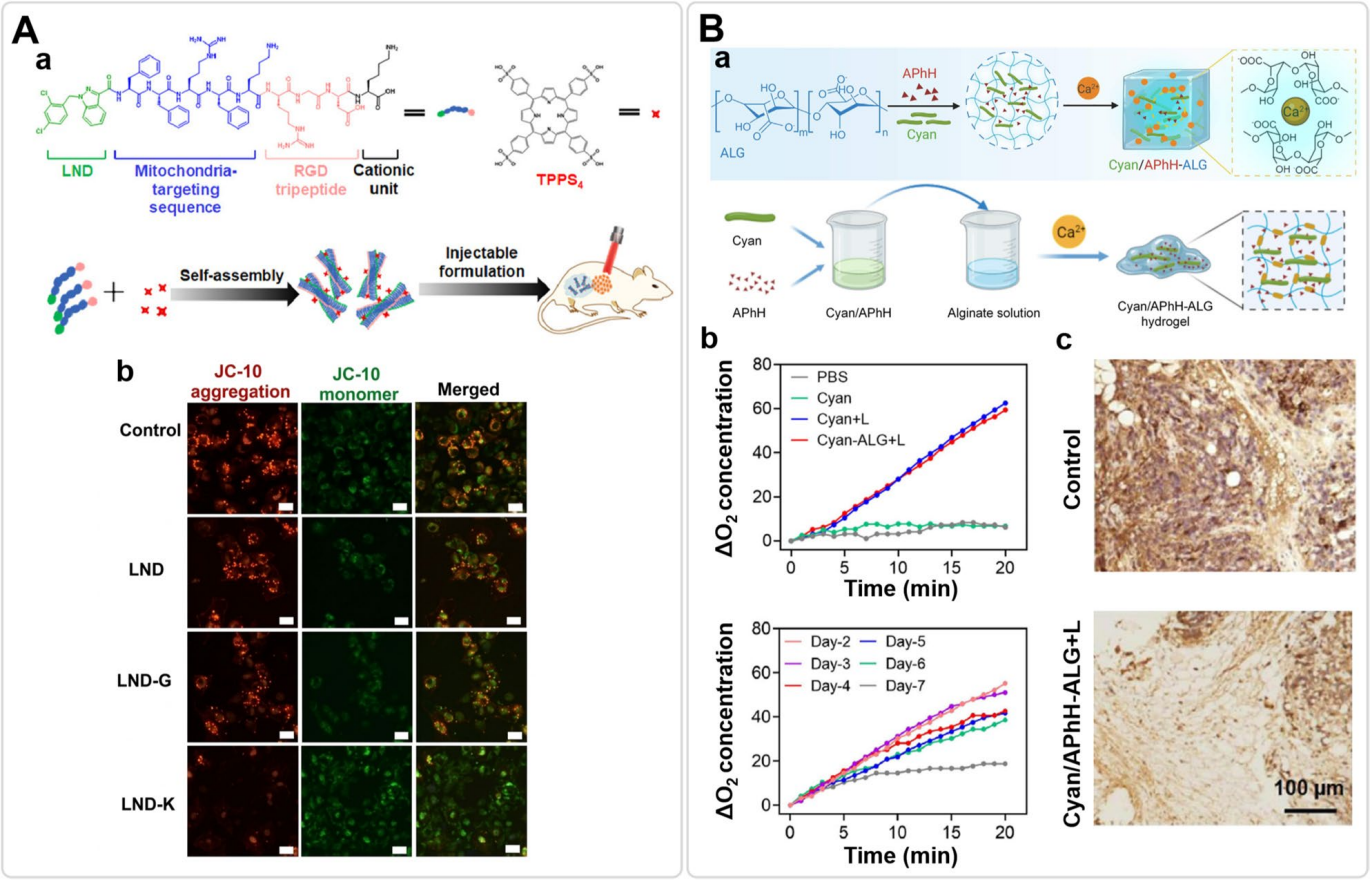
Functional hydrogel depots to support photodynamic and oxidative therapies. **A** Mitochondria-targeted peptide hydrogel. **A** Conceptual schematic of lonidamine–peptide conjugates (LND-K) and TPPS4 co-assembly into a nanofibrillar hydrogel, illustrating the co-assembly logic for dual-targeting therapy. **b** Representative confocal fluorescence images (JC-10 staining) tracking mitochondrial membrane potential (ΔΨ_m_). Control cells, or those treated with pure LND solution or non-targeting LND-G control, display bright red fluorescence (JC-10 aggregates, high ΔΨ_m_). Conversely, LND-K hydrogel treatment induces massive ΔΨ_m_ loss, marked by an apparent red-to-green fluorescence shift. Adapted from Ref [104]. Copyright 2022, Elsevier Ltd. **B** Photosynthetic alginate hydrogel for hypoxia relief. **a** Schematic illustration of the synthesis of Cyan/APhH-ALG hydrogel. **b** Dissolved O^2^ traces indicate sustained photosynthetic oxygen production. **c** Immunofluorescence of tumor sections shows reduced HIF-1α (green) after light exposure. Adapted from Ref [45]. Copyright 2025, Wiley-VCH GmbH

Beyond cutting off fuel supplies, directly targeting mitochondria has been shown in preclinical studies to interrupt bioenergetic plasticity. The double membrane of mitochondria is highly restrictive and the inner membrane favors cation handling, limiting many cargos [102]. Advanced hydrogels have been engineered to release “mitochondriotoxic” agents that disrupt mitochondrial function. One example is the self-assembling peptide hydrogel reported by Wu et al., which incorporates a mitochondria-targeting lonidamine derivative (LND-K) (Fig. 5A). In vitro and in vivo assays indicated that the peptides assemble into nanofibrillar networks and accumulate in mitochondria following a chaperone-like structural shift [103]. Functionally, this was observed to collapse mitochondrial membrane potential (ΔΨ_m_) and deplete ATP, potentially lowering the threshold for synergistic photodynamic therapy in these models [104].

A related step toward drug-resistant disease comes from a radiation-triggerable bioreactor co-delivering a therapeutic plasmid and β-lapachone [105]. This formulation was reported to contribute to mitochondrial dysfunction via arginine metabolism disruption and ROS generation, subsequently triggering immunogenic necroptosis and reducing stemness-related resistance in preclinical settings. Significantly, resistant cancer stem cells (CSCs) have been observed to rely more heavily on oxidative phosphorylation (OXPHOS), which potentially renders them selectively vulnerable to mitochondrial disruption [106]. Consistent with this logic, decreased expression of stemness regulators such as Nanog and SOX2 has been reported in these experimental settings [107], suggesting that mitochondrial targeting may tighten the limits of tumor bioenergetic plasticity.

### Ameliorating the hypoxic microenvironment: oxygen-generating and carrying systems

#### In situ oxygen generation: converting waste to fuel

To overcome hypoxia-induced resistance, hydrogel strategies have moved beyond simple oxygen carriers toward in situ oxygen generation. Hypoxia, however, is not just a supply issue; it drives proteomic and transcriptional rewiring, largely through Hypoxia-Inducible Factors (HIFs), which have been shown in various settings to contribute to multidrug resistance [108]. Traditional manganese dioxide (MnO₂)-based schemes that convert endogenous H₂O₂ often run into a basic constraint: the peroxide pool is finite in many tumors [109]. While oxygenation is a cornerstone of hypoxia relief, it may play a dual role in tumor progression. If oxygen supply is not properly synchronized with ROS-dependent modalities (e.g., photodynamic or chemodynamic therapy) or immune-activating agents, improved oxygenation can potentially restore the proliferative capacity of aerobic tumor cells [110–112]. Furthermore, the efficacy of in situ oxygenation is fundamentally constrained by diffusion–consumption dynamics and the functional lifetime of the oxygen-generating depot—ranging from transient bursts (minutes) to sustained release (days) depending on the catalytic chemistry and matrix permeability [113]. Importantly, oxygen delivery is spatially limited by diffusion gradients in tumor tissue, typically extending only ~ 100–200 μm from functional microvessels, beyond which oxygen consumption by tumor cells can rapidly establishes hypoxic zones [114, 115].

Recent work therefore leans on self-sustaining designs. One example is an “AND”-gated living hydrogel incorporating photosynthetic cyanobacteria (Fig. 5B). Under light, photosynthesis continuously generates oxygen; that oxygen then serves as a required cofactor for tyrosinase-catalyzed prodrug activation, closing the loop into a preclinical ferroptosis cascade observed in melanoma models [45]. For the longer oxygenation windows demanded by fractionated regimens, Zhang et al. integrated calcium peroxide (CaO₂) with catalase-mimetic nanoceria, achieving sustained oxygen release for up to 7 days in vivo and improving outcomes across repeated photodynamic therapy (PDT) sessions [116]. To work around light penetration limits, persistent luminescence nanoparticles (PLNPs) have been embedded in hydrogels. In preclinical models, these systems relieve hypoxia catalytically while banking excitation energy to support deep-tissue, laser-free PDT, and they have been shown to potentially blunt indoleamine 2,3-dioxygenase (IDO)-linked immunosuppression by downregulating the HIF-1α/IDO axis [117].

#### Artificial oxygen carriers: perfluorocarbons and hemoglobin mimics

When endogenous H₂O₂ is insufficient to sustain catalytic oxygen generation, hydrogels can be engineered to bring in oxygen from outside and move it toward the tumor core [118]. Perfluorocarbons (PFCs)—chemically inert liquids with exceptionally high oxygen solubility—function as oxygen sinks/reservoirs, releasing dissolved O₂ largely by passive diffusion [46].

Not all “carriers” are passive, though. Metal–organic frameworks (MOFs) can be engineered as high-capacity oxygen loaders with additional functionality; for example, a fluorinated zirconium MOF (69-L2@F) couples oxygen storage with intrinsic photosensitization, which has been reported to help disrupt hypoxia-linked resistance loops in preclinical models [119]. To narrow the gap between synthetic carriers and native blood, hydrogel-encapsulated hemoglobin-based oxygen carriers (HBOCs) have also been pursued. Hierarchical structural engineering of porous MOFs has been used to stabilize Hb and extend its usable lifetime in circulation [120]. Meanwhile, theoretical analyses suggest that oxygen affinity matters, and the *P*_50_ must be tuned for preferential unloading in hypoxic niches [121]. These Hb-laden depots frequently provide multifunctional benefits beyond oxygenation alone. They have been shown to supply Fe²⁺ as a catalytic node for chemodynamic reactions [122], act as endogenous sonosensitizers to boost ROS generation in preclinical settings [123], and can form oxygen-rich local immune niches that have been shown in preclinical observations to help sustain chimeric antigen receptor (CAR) T-cell activity in an immunosuppressive TME [124].

### Practical design rules for metabolic hydrogel depots

To facilitate the clinical translation of metabolic interventions, several design imperatives are emerging from recent preclinical investigations [125, 126]. First, maintaining a precise pO_2_ window, typically 10–30 mmHg, is critical for destabilizing HIF-1α-associated resistant phenotypes without excessively stimulating the proliferative capacity of aerobic tumor cells [127]. This range aims to balance the need to sensitize oxidative therapies with the risk of inadvertently supporting tumor metabolic demands. Second, evidence indicates that release kinetics should be ideally sustained over several days to better accommodate the temporal demands of fractionated radiotherapy compared to transient oxygen bursts [116]. Third, the incorporation of intrinsic buffering capacity, such as through CaCO_3_-based materials, is hypothesized to neutralize acidic byproducts and support the metabolic fitness of infiltrated immune effectors while maintaining enzyme stability [128]. Finally, the integration of real-time monitoring of pO_2_, pH, or lactate—facilitated by embedded biosensors or image-guided readouts—presents a promising avenue for optimizing the sequencing of multi-modal combinations. Such feedback-driven systems may allow for more precise timing of secondary interventions once the metabolic barrier has been appropriately modulated [129].

## Subverting cellular defenses: efflux evasion and intracellular navigation

**Fig. 6:**
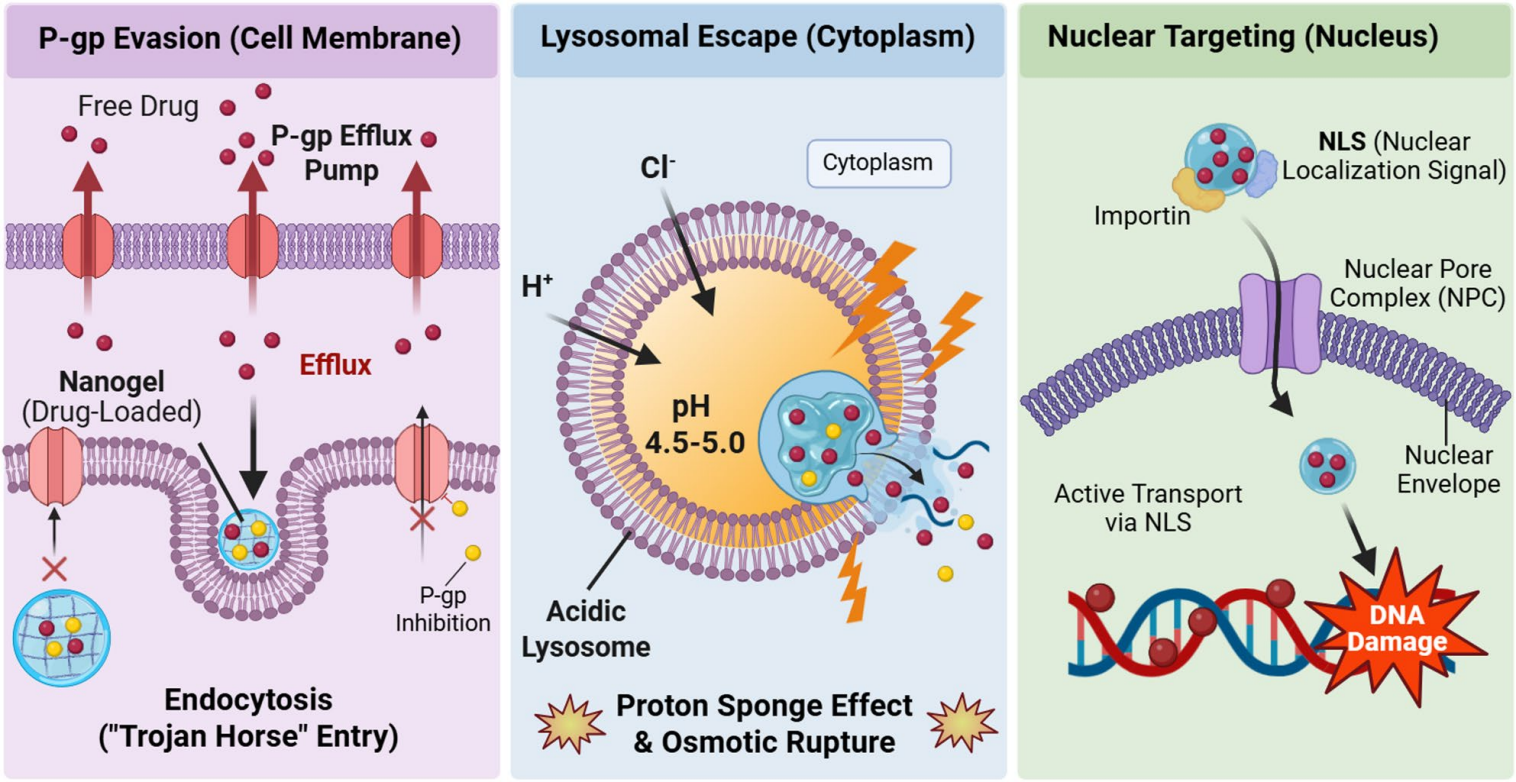
Nanogel-enabled routes to bypass cellular multidrug resistance (MDR) barriers and improve subcellular delivery. Free drugs are direct substrates of membrane P-glycoprotein (P-gp) efflux pumps and are readily exported. Nanogels preferentially enter cells via endocytosis, limiting immediate exposure to plasma-membrane efflux. After internalization, endolysosomal trapping is reduced through designs that promote vesicle escape, including proton-sponge–driven swelling and osmotic rupture, enabling cytosolic release. Nuclear-targeting ligands (NLS) can further recruit importins to support transport across the nuclear pore complex, facilitating delivery to nuclear targets in resistant cells. Created with BioRender.com

Even after overcoming the dense stromal barrier and surviving hypoxic stress, therapeutics still face a final, cell-level barrier [5]. This defense system functions as a two-step barrier with two main checkpoints: the plasma membrane, dominated by MDR efflux pumps, notably P-gp, that actively expel therapeutic agents, and the intracellular milieu, where acidic endolysosomal compartments and organelles can sequester or enzymatically degrade cargo [30, 130]. Conventional free drugs, which largely rely on diffusion, are frequently intercepted at these interfaces, potentially resulting in intracellular exposure below the therapeutic window. Nanogels address this by modulating both the entry route and the intracellular trafficking pathway [131]. Following endocytic uptake, stimuli-responsive designs can promote endosomal escape and guide subcellular trafficking, which has been shown in preclinical models to reduce efflux and improve intracellular delivery (Fig. 6) [132, 133].

### Circumventing efflux pumps: nanogel-mediated endocytic bypass and pump suppression

**Fig. 7:**
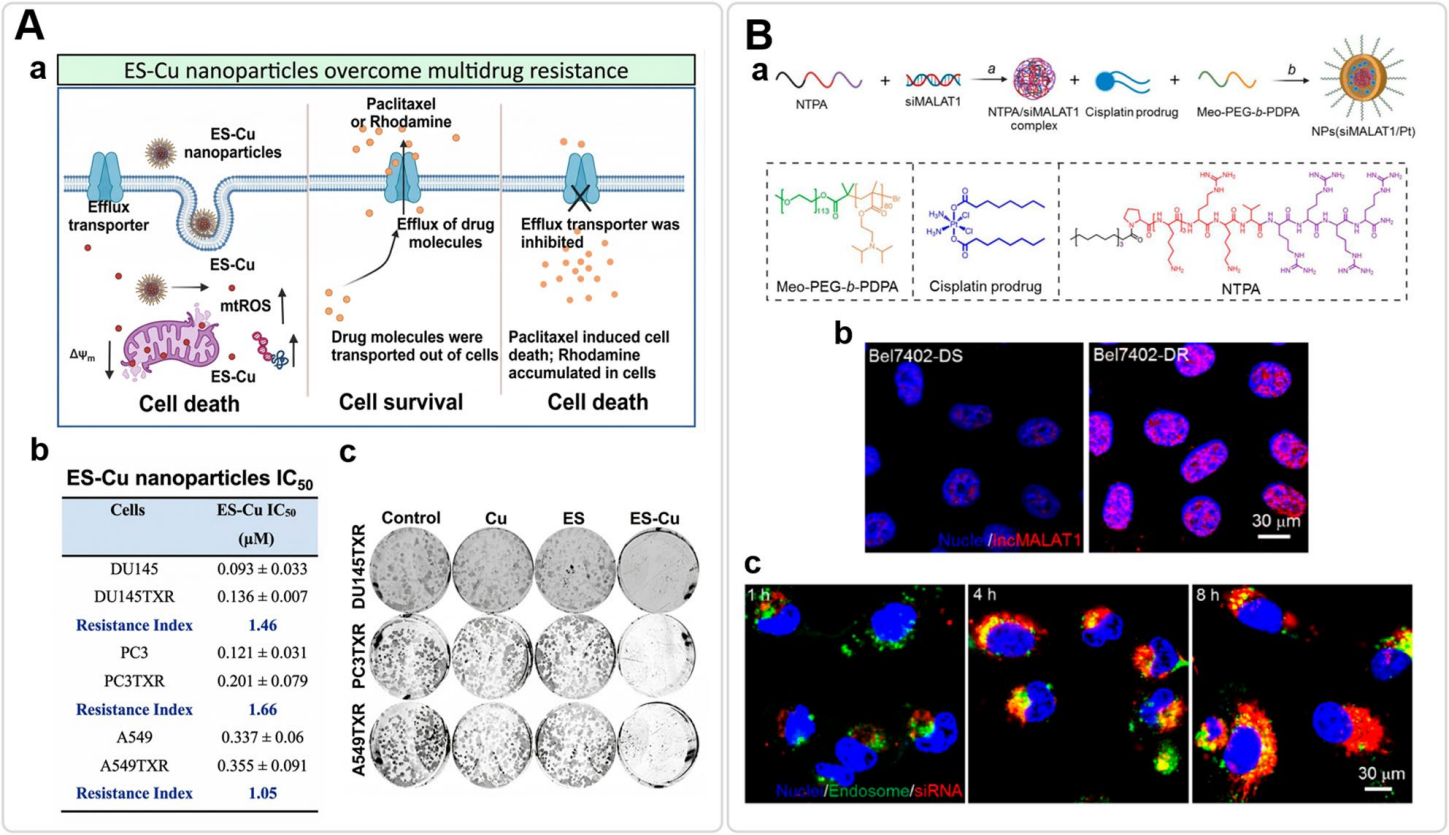
Nanogel strategies to bypass efflux and enable subcellular delivery. **A** Endocytic bypass of P-gp using ES–Cu micellar nanoparticles. **a** Schematic mechanism of ES-Cu nanoparticles overcoming multidrug resistance (MDR). **b** Comparable cytotoxicity in drug-sensitive and multidrug-resistant cell lines (resistance index ≈ 1). **c** Suppressed long-term colony formation in resistant cells, consistent with functional efflux evasion. Adapted with permission from Ref [50]. Copyright 2024, American Chemical Society. **B** Nuclear delivery for lncRNA silencing. **a** Endosomal pH-responsive nanoplatform co-delivers siMALAT1 and cisplatin; proton-sponge escape enables cytosolic release, and NTPA-mediated nuclear transport promotes nuclear accumulation to silence lncMALAT1 and restore cisplatin sensitivity. **b** FISH confirms depletion of nuclear lncMALAT1 in resistant cells. **c** Confocal imaging and nuclear signal quantification show progressive endosomal escape and nuclear accumulation over 8 h. Adapted with permission from Ref [58]. Copyright 2024, Wang et al. (Open Access)

One of the primary mechanisms contributing to MDR is the overexpression of ABC transporters, particularly P-gp, which actively extrudes small-molecule drugs from the cytosol [134]. Nanogels have been reported to circumvent this barrier by altering the mechanism of cellular entry. Unlike free drugs that rely on passive diffusion and are readily recognized by membrane-bound efflux pumps, nanogels are primarily internalized via endocytic pathways. This vesicular uptake can sequester the payload away from P-gp recognition domains on the plasma membrane [30, 135]. Beyond this steric evasion, excipients within the nanogel matrix can directly inhibit transporter function. D-α-tocopheryl polyethylene glycol 1000 succinate (TPGS) is frequently utilized as a biological response modifier; it has been shown to inhibit P-gp ATPase activity and increases membrane fluidity [136]. For instance, in preclinical studies, TPGS-based nanogels delivering an Elesclomol–Copper complex were observed to enter cells via endocytosis and reduce P-gp-mediated export, subsequently inducing mitochondrial oxidative stress (cuprotosis) in drug-resistant cells where the free drug showed limited activity (Fig. 7A) [50]. In practice, this coordinated sequestration and inhibition strategy combines vesicular trafficking with biochemical stress to attenuate the MDR phenotype.

### Overcoming subcellular barriers: lysosomal escape and nuclear targeting

Following endocytic uptake, nanogels are required to traverse a complex intracellular landscape shaped by acidic sequestration and organelle transport barriers [137]. To circumvent the endolysosomal barrier (pH ≈ 5.0), advanced nanogels often rely on “proton sponge”-mediated swelling: ionization of internal buffering groups has been reported to drive rapid volumetric expansion that can mechanically rupture the vesicle membrane [53]. Alternatively, dynamic charge-reversal chemistries have been shown in preclinical studies to offer a more direct route. For example, PH-sensitive benzoic imine cross-linkers cleave in the acidic milieu, shifting the surface charge from negative to positive and subsequently destabilizing the lipid bilayer [52].

Once in the cytosol, the nuclear envelope serves as a significant size-exclusion barrier (pore size ≈ 9–39 nm). To bypass this limitation, nanogels have been engineered for hierarchical disassembly. For instance, it has been demonstrated in preclinical investigations that some systems use a cleavable PEGylated shell that detaches under intracellular cues, thereby reducing steric bulk and enhancing nuclear access for macromolecular payloads such as clustered regularly interspaced short palindromic repeats–Cas9 (CRISPR–Cas9) [51]. This passive size reduction is often paired with active transport: nanogels functionalized with nucleus-targeting amphiphilic peptides (NTPA) have been shown to facilitate the translocation of cargos through the nuclear pore complex, enabling the silencing of nuclear-localized long noncoding RNAs (lncRNAs) and potentially contributing to the attenuation of drug resistance as observed in preclinical models (Fig. 7B) [58].

## Awakening the immune system: spatiotemporal modulation of the cancer-immunity cycle

**Fig. 8:**
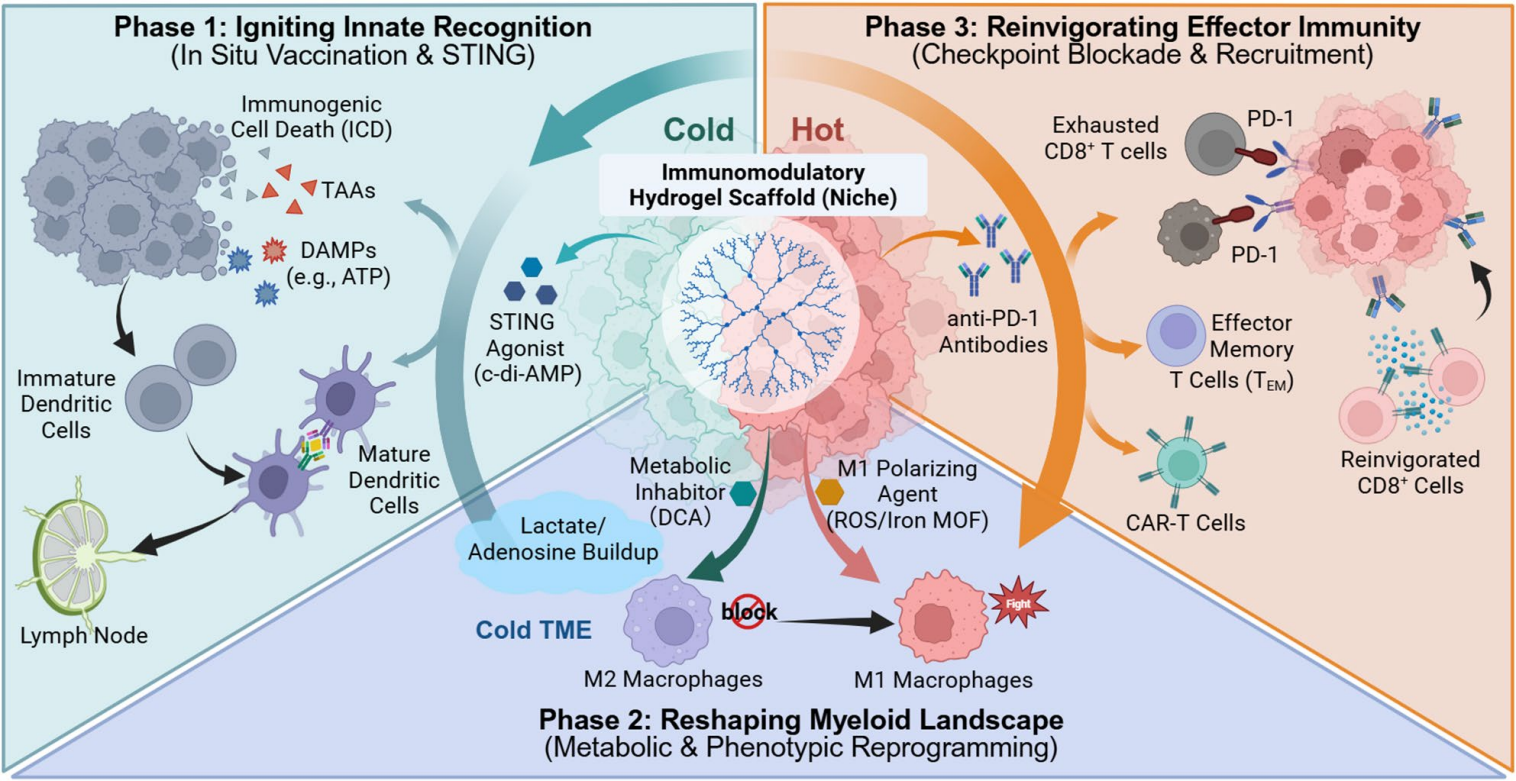
Hydrogel scaffold–mediated reprogramming of the tumor immune microenvironment (TIME). The schema depicts a three-step restart of the cancer–immunity cycle, pushing a “cold” tumor microenvironment (TME) toward a “hot” state. Phase 1: Innate ignition. The hydrogel releases STING agonists such as c-di-AMP, alongside immunogenic cell death (ICD)–derived tumor-associated antigens (TAAs) and damage-associated molecular patterns (DAMPs), promoting dendritic-cell maturation and trafficking to draining lymph nodes. Phase 2: Myeloid reset. Metabolic inhibitors (for example, DCA) limit lactate/adenosine accumulation, while M1-polarizing cues (for example, ROS/iron MOF) drive M2-to-M1 repolarization. Phase 3: Effector reinforcement. Local anti–PD-1 release sustains checkpoint blockade and restores CD8⁺ T-cell activity. In parallel, the remodeled niche supports effector memory T cells and cell therapies such as CAR T cells, maintaining a productive antitumor immune loop. Created with BioRender.com

While overcoming intracellular barriers facilitates direct cytotoxicity, achieving durable tumor control requires the restoration of systemic antitumor immunity. Yet the so-called cancer–immunity cycle is often impaired in solid tumors, leaving an immunologically “cold” microenvironment marked by poor antigen presentation and entrenched immunosuppression [138, 139]. In this setting, hydrogel scaffolds have evolved from passive delivery vehicles and are increasingly used as local, programmable immunomodulatory platforms. A recurring theme across recent studies is timing and sequence: local in situ vaccination to ignite innate recognition [55, 140], metabolic rewiring of lactate/adenosine stress that mitigates myeloid suppression [47], and sustained checkpoint blockade to alleviate exhaustion in effector cells (Fig. 8) [141–143]. These interventions shift the balance of the tumor microenvironment away from tolerance and toward immune pressure on malignant cells.

### Igniting innate immunity: in situ vaccination and STING activation

#### In situ vaccination through immunogenic cell death

Promoting the development of an endogenous tumor vaccine is dependent on the co-release of tumor-associated antigens (TAAs) and danger cues, such as damage-associated molecular patterns (DAMPs) to prime antigen-presenting cells. Hydrogel scaffolds are well-suited for in situ vaccination because they maintain immunogenic cell death (ICD) inducers at the target site [144, 145]. For instance, Fe-doped carbon dot–incorporated hydrogels generate localized hyperthermia and alkyl radicals under NIR irradiation, inducing a cascade of ICD events—calreticulin exposure and ATP release—that promote dendritic cell (DC) maturation and subsequent CD8 + T-cell infiltration [54]. To further enhance these effects, some designs incorporate DC-recruiting factors. A hydrogel microsphere vaccine releasing Fms-like tyrosine kinase 3 ligand (FLT3L) and CD40 ligand (CD40L) was reported to recruit type 1 conventional dendritic cells (cDC1) into the tumor bed and support their migration to draining lymph nodes, thereby initiating antigen cross-presentation and transforming non-immunogenic (“cold”) pancreatic tumors into highly inflamed (“hot”) phenotypes [146].

#### Enhancing innate sensing: STING pathway activation

Antigen release is necessary, but its immunological impact is often limited without a strong innate trigger. The cyclic GMP–AMP synthase (cGAS)–STING axis is a key bridge [147, 148], yet systemic STING agonists are limited by rapid clearance and dose-limiting toxicity [149]. Hydrogel reservoirs optimize pharmacokinetics by enabling sustained, intratumoral dosing. Wang et al. demonstrated that a self-assembling peptide hydrogel releasing the STING agonist cyclic di-AMP (c-di-AMP) prolonged intratumoral exposure, modulating the microenvironment from immunosuppressive to immunostimulatory and promoting long-term immunological memory [57]. More recently, environmentally responsive hydrogels co-delivering c-di-AMP and the vascular normalizer RRx-001 were designed to coordinate vascular remodeling with immune activation: RRx-001 improves vascular function and facilitates leukocyte infiltration, while c-di-AMP engages STING signaling. In preclinical models, this pairing showed synergistic control of established tumors [56].

### Reconfiguring the myeloid compartment: metabolic and phenotypic reprogramming

#### Metabolic intervention: attenuating lactate and adenosine barriers

The accumulation of oncometabolites—especially lactate and adenosine—creates a metabolic environment that inhibits effector T-cell function while promoting a suppressive myeloid phenotype [150, 151]. To mitigate Warburg-linked immunosuppression, Lu et al. developed a chrono-controlled hydrogel that releases dichloroacetate (DCA) in sequence. DCA shifts tumor metabolism from glycolysis toward oxidative phosphorylation, reducing intratumoral lactate. That shift results in two primary effects: it restricts glucose utilization by cancer cells and restores metabolic homeostasis in T cells, rendering tumors more susceptible to mitochondrial injury and immune-mediated pressure [47].

Adenosine is another critical component of this metabolic challenge. Targeting the adenosinergic axis remains essential; biomaterials that modulate adenosine signaling—by limiting its generation or blocking its receptors—can reduce macrophage polarization toward the M2 state, thereby inhibiting a significant pathway of immune escape [152–154].

#### Modulating myeloid plasticity: from depletion to repolarization

Beyond metabolic control, targeting the myeloid compartment directly can be pivotal for enhancing the responsiveness of non-immunogenic tumors [155]. One approach involves the depletion of suppressive populations. For example, an alginate-based hydrogel releasing the colony-stimulating factor 1 receptor (CSF-1R) inhibitor pexidartinib (PLX3397) depletes TAMs, facilitating cytotoxic T-cell infiltration and improving the performance of anti-PD-1 therapy in vivo [156].

**Fig. 9:**
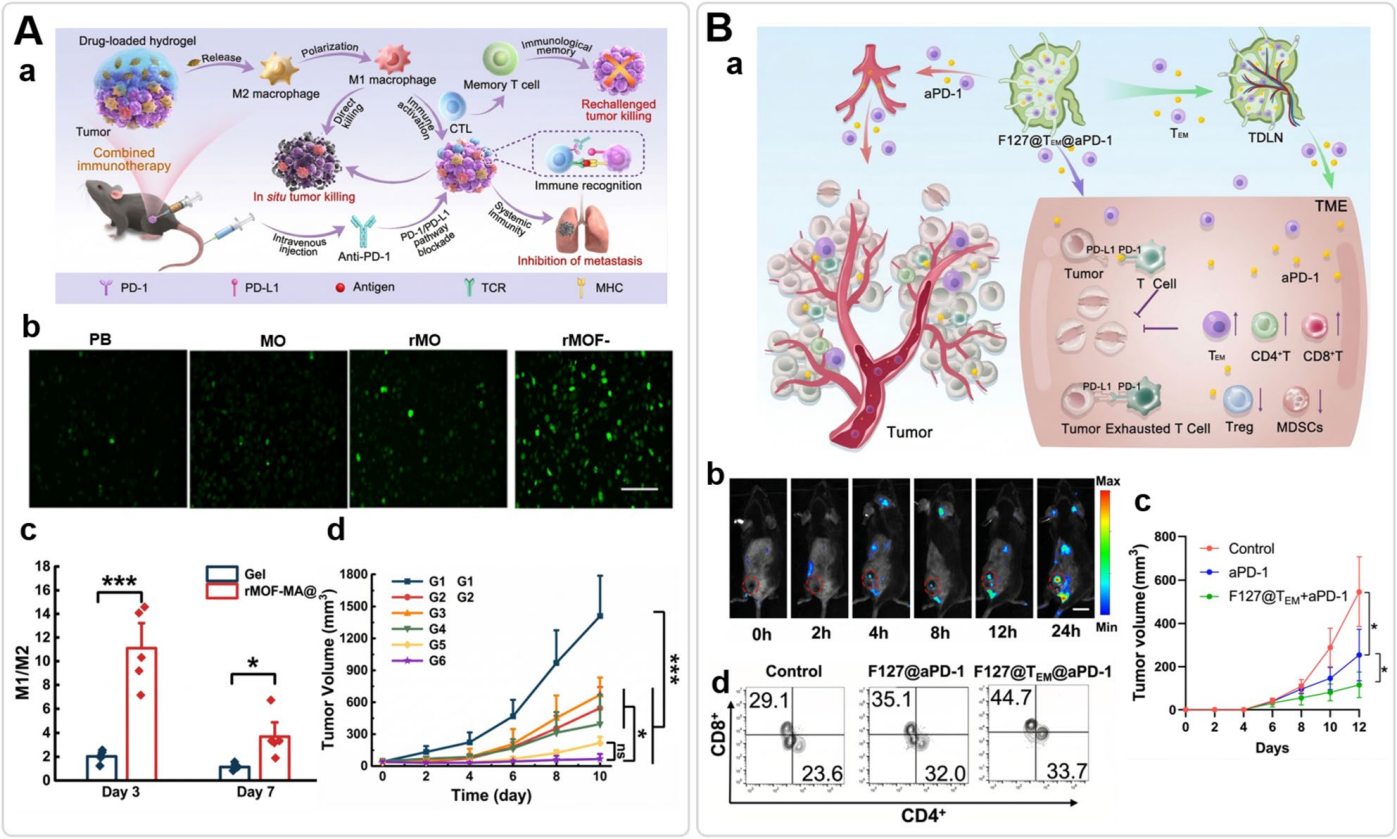
Multifunctional hydrogel depots for cancer immunotherapy. **A** Metal–organic framework (MOF)-based adhesive hydrogel for macrophage polarization. **a** Conceptual schematic illustrating the applications of R848@rMOF-MA@Gel for localized combination cancer immunotherapy. **b** Representative fluorescence images of treated bone marrow-derived macrophages (BMDMs), illustrating significantly increased intracellular reactive oxygen species (ROS) production (green) in the rMOF-MA group compared to control treatments (Scale bar = 200 μm). **c** Mechanistic analysis of macrophage polarization in vivo, quantitative analysis of infiltrating M1/M2 macrophage ratios on day 3 and day 7, demonstrating the effective induction of pro-inflammatory phenotypes by the rMOF-MA@Gel. **d** Therapeutic efficacy in vivo, showing tumor growth curves of B16F10 melanoma-bearing mice treated with various formulations combined with anti-PD-1, demonstrating the feasibility of the hydrogel-mediated localized combination immunotherapy. Reproduced with permission from Ref [141]. Copyright 2025, American Chemical Society. **B** Lymph node–targeting hydrogel for T-cell and checkpoint blockade delivery. **a** Conceptual schematic illustrating a thermosensitive F127 hydrogel depot co-loading effector memory T cells (T_EM_) and anti-PD-1 (αPD-1), designed to target both tumor-draining lymph nodes (TdLN) and the tumor microenvironment (TME) (**b**) Maximized representative in vivo imaging tracks, illustrating the progressive migration of hydrogel-released T_EM_ cells toward the tumor site over 24 h. **c** In vivo antitumor activity, presenting tumor growth curves of various treatment groups, demonstrating significant tumor growth inhibition by the gel-mediated combination therapy. **d** Mechanistic analysis of TME remodeling, presenting representative flow cytometry plots of infiltrated CD4^+^ and CD8^+^ T cells in tumors, demonstrating the effective induction of pro-inflammatory phenotypes that dismantling of the immune barrier by the gel-mediated therapy. Reproduced with permission from Ref [142]. Copyright 2024, Elsevier

An alternative strategy focuses on functional reprogramming rather than depletion. Iron-based metal–organic framework (MOF) hydrogels can act as local depots that raise intracellular ROS and iron burden in macrophages, triggering a phenotypic switch from protumoral M2 to antitumoral M1 states (Fig. 9A) [157–159]. Paired with the immune adjuvant R848, this strategy reorganizes local inflammation and has been used to suppress post-surgical recurrence in preclinical settings [141]. Finally, ROS-responsive scaffolds carrying PI3Kγ inhibitors (e.g., IPI549) can disrupt myeloid-driven immunosuppressive microenvironments, creating a therapeutic window for checkpoint blockade in otherwise resistant tumors [160].

### Reinvigorating effector immunity: synergistic checkpoint blockade and overcoming immune exclusion

#### Sustained checkpoint blockade: biomimetic scaffold strategies

Systemic immune checkpoint blockade (ICB) is often compromised by low intratumoral accumulation and immune-related adverse events. Hydrogel scaffolds address this by acting as localized biomimetic immunological niches, maintaining checkpoint pathways under sustained inhibition while recruiting effector cells [161–163]. For instance, Cui et al. engineered a biomimetic F127 hydrogel depot co-delivering PD-1 inhibitors and effector memory T cells (T_EM_) (Fig. 9B). This platform not only maintained therapeutic antibody concentrations in tumor-draining lymph nodes (TdLN), but also continuously supplied the TdLN with T_EM_ cells. Consequently, priming and infiltration proceed as a sustained cycle rather than a transient event, modulating “cold” tumors toward “hot” phenotypes [142].

Synergy is further observed when immune-regulating metal–organic frameworks (MOFs) are integrated into tissue-adhesive hydrogels to establish a locally inflammatory microenvironment (Fig. 9A). ROS generated by iron-based MOFs promotes macrophages repolarization which, together with sustained release of R848 and ICB antibodies, helps reversing CD8 + T cells exhaustion and reducing post-surgical recurrence in preclinical studies [141].

#### Reversing immune exclusion: in situ programming of adoptive cell therapy

Adoptive cell therapy (ACT), particularly CAR T cells, is limited in solid tumors due to physical exclusion and functional exhaustion. Advanced hydrogels are now being designed as local activation and expansion platforms to overcome these barriers [164, 165]. A practical upside of localizing this process is safety: by confining activation and expansion within the engineered niche, systemic toxicities such as cytokine release syndrome (CRS) can be mitigated [166, 167]. In this vein, He et al. developed injectable hydrogel microspheres functionalized with anti-CD3/anti-CD28 antibodies and IL-7/IL-15–loaded nanoparticles. The scaffold mimics the costimulatory mechanisms of secondary lymphoid organs, delivering co-stimulatory and survival cues that locally expand and enhance the activity of tumor-infiltrating lymphocytes (TILs), notably, ~ 95% inhibition of primary osteosarcoma growth was reported in preclinical mouse models [168].

Moving beyond cell delivery, Zhu et al. introduced a supramolecular hydrogel for in situ CAR T programming. By delivering plasmid CAR (pCAR) via CD3-targeted cationic polymers within the gel, endogenous T cells are transfected on site. The resulting de novo CAR T cells—supported by hydrogel-mediated release of inflammatory cytokines (IL-2, IFN-γ)—can penetrate dense stroma and have been shown to facilitate tumor regression in experimental models [169].

## Future perspectives: bridging the translational gap

While hydrogel-based immunotherapeutic strategies have demonstrated encouraging activity in overcoming physical barriers within the tumor microenvironment and restoring antitumor immune activity in preclinical tumor models (representative strategies and their translational profiles are summarized in Table 2), their progression toward clinical application remains challenging, with substantial attrition often described as the translational “Valley of Death” [170–172]. Moving from preclinical proof-of-concept to clinical utility will likely require a shift from a predominantly material-centric innovation model toward a more translation-oriented framework that incorporates considerations of manufacturability, regulatory requirements, and clinical feasibility [173–175]. Key bottlenecks include challenges in manufacturing scale-up and Chemistry, Manufacturing, and Controls (CMC) complexity, uncertainties regarding the long-term biological fate and biodegradation of implanted materials [176], and substantial inter-patient heterogeneity in immune responses and tumor microenvironmental states [177, 178].

**Table 2:** Comparative analysis of emerging hydrogel platforms (2023–2026): a clinical and engineering roadmap

Strategy Category	Representative System	Synergistic Mechanism	Route of Administration	Est. Degradation Time	Evidence Level	Translational Pros & Cons	Ref.
Living Materials	Cyanobacteria-driven living hydrogel	Metabolic + Physical: Photosynthetic O_2_ generation relieves hypoxia and induces ferroptosis.	Peritumoral (Melanoma specific)	~ 21 Days (Alginate matrix)	In vivo (Orthotopic melanoma)	Pros: Self-sustaining O_2_ supply. Cons: Biosafety of live bacteria; light penetration depth limit.	[45]
Nanozyme Cascades	LDO-embedded composite bioreactor	Metabolic + Cellular: Glucose starvation coupled with ROS burst (Chemodynamic Therapy).	Intratumoral	~ 30 Days (slow resorption)	In vivo (Syngeneic & 3D organoids)	Pros: High chemical stability; no resistance induction. Cons: Long-term metal toxicity risk; synthesis complexity.	[95]
Genetic Programming	Supramolecular pCAR-depot	Immune + Cellular: In situ transfection of T cells to generate CAR-T cells locally.	Peritumoral (Adjacent to solid tumors)	10–14 Days (Reversible crosslinking)	In vivo (Orthotopic solid tumors)	Pros: “Off-the-shelf” CAR-T; bypasses manufacturing. Cons: Human transfection variability; gene safety.	[169]
Microenvironment Normalizers	RRx-001/STING responsive gel	Physical + Immune: NO release normalizes vessels (lowers IFP); STING activates innate immunity.	Intratumoral / Peritumoral	~ 7 Days (pH-responsive)	In vivo (Tumor rechallenge models)	Pros: Directly addresses IFP barrier; enhances CD8 + infiltration. Cons: Precise NO dose-dependency is critical.	[56]
Metabolic Interferers	SeqGel (PEG-based polypeptide)	Metabolic + Immune: DCA switches glycolysis to OXPHOS; depletes lactate to reverse T cell exhaustion.	Peritumoral (Repeated administration)	< 48 h (Tunable bio-cleavage)	In vivo (TNBC metastasis models)	Pros: Bioactive backbone; addresses T cell fitness. Cons: High DCA dose requirement; peptide cost.	[47]
Mechanomodulators	GHAM (Magneto-softening gel)	Physical + Cellular: Dynamic mechanical rescue via wireless YAP downregulation and drug sensitization.	Intratumoral / Post-resection	> 60 Days (Stable magnetic niche)	Ex vivo / In vivo (Mechanobiological study)	Pros: Wireless, non-invasive, reversible control. Cons: Requires external equipment; depth limit.	[42]

*Abbreviations*: *CAR-T* Chimeric antigen receptor T-cell, *CoMnFe* Cobalt-manganese-iron, *DCA* Dichloroacetate, *GOx* Glucose oxidase, *HCC* Hepatocellular carcinoma, *IFP* Interstitial fluid pressure, *LDO* Lactate oxidase, *NO* Nitric oxide, *OXPHOS* Oxidative phosphorylation, *ROS* Reactive oxygen species, *STING* Stimulator of interferon genes, *YAP* Yes-associated protein

### Translational engineering: from manufacturing discipline to injection science

The clinical success of multifunctional hydrogel depots is closely linked to a robust Chemistry, Manufacturing, and Controls (CMC) profile. The transition from laboratory-scale synthesis to Good Manufacturing Practice (GMP) production is frequently constrained by the structural complexity of these platforms. A recent Nature Nanotechnology consensus highlighted the DELIVER framework (Design, Engineering, Life-cycle, Interactions, Value, Efficacy, and Regulatory) as a strategy for identifying translational risks early in development [179]. In particular, the CMC complexity of multifunctional constructs—such as messenger RNA (mRNA)-loaded lipid–hydrogel hybrids or multi-component nanogel depots—remains a major bottleneck. Emerging manufacturing approaches, including microfluidic and automated synthesis platforms, are increasingly employed to improve reproducibility and reduce batch-to-batch variation during scale-up [180–182]. From a regulatory perspective, Quality by Design (QbD) frameworks provide a systematic strategy for linking critical quality attributes (CQAs)—including parameters such as mesh size uniformity and cross-linking density—to clinically relevant safety and performance outcomes [28, 183–186].

Moving from manufacturing to clinical administration, the success of a local depot is equally contingent upon what may be termed “injection science.” Early clinical attempts, such as the paclitaxel-loaded OncoGel, highlighted the challenge of achieving consistent therapeutic outcomes despite promising preclinical profiles [187, 188]. In contrast, the successful translation of the FDA-cleared SpaceOAR hydrogel system underscores that clinical viability depends not only on material safety but also on standardized operator training and quality assurance (QA) to prevent complications such as unintended tissue infiltration [189]. Mechanistically, intratumoral retention and spatial coverage are fundamentally constrained by the interplay between hydrogel rheology—specifically yield stress and gelation kinetics—and the tumor’s mechanical environment. In practice, high IFP and stromal heterogeneity can trigger reflux along the needle track or lead to non-uniform filling of necrotic cavities [190–192]. Therefore, regulatory progress and Quality by Design (QbD) approaches must increasingly link critical quality attributes (CQAs) to these practical delivery constraints to ensure reproducible efficacy in clinical oncology.

### Long-term biocompatibility: addressing the foreign body response

While acute toxicity is routinely evaluated, the long-term immunological fate of implanted hydrogels remains a relative blind spot and is increasingly recognized as a context-dependent and actively regulated outcome shaped by the interplay between material chemistry, mechanical properties, degradation kinetics, and the local immune context. Distinguished from the localized sequestration response observed with traditional rigid implants, which typically induce a well-defined and dense isolating fibrous capsule, certain injectable hydrogels exhibit a “dispersion” pattern that leads to more complex material–tissue integration and a broader induction of specialized immunosuppressive myeloid niches. Recent single-cell RNA sequencing studies of long-term hydrogel exposure—particularly in certain non-degradable systems such as polyacrylamide hydrogel (PAAG)—suggest that prolonged stimulation may elicit a chronic foreign body response (FBR), which is associated with a shift of the local milieu toward a more immunosuppressive state, accompanied by fibrosis and macrophage sequestration, and has been implicated in tumor-promoting processes [193]. This inflammation-associated fibrosis not only imposes a secondary physical barrier to drug penetration but may also establish a mechanically protective niche that re-engages mechanotransduction-linked resistance programs [194, 195], highlighting the unresolved challenge of mitigating foreign body responses while preserving, or potentially enhancing, antitumor immunity within the tumor microenvironment. For nanogel systems, bio–nano interactions—particularly liver sequestration and clearance—are equally consequential for limiting systemic immunotoxicity and off-target accumulation [32]. In parallel, protein corona formation in circulation can rewrite nanogel biological identity, prompting macrophage recognition and premature clearance before the carrier reaches the tumor [196–198]. Future designs should prioritize biodegradable backbones with immunologically inert, or pro-resolving, degradation products to support safety across extended dosing windows.

### The AI-organoid nexus: accelerating predictive design

The integration of Artificial Intelligence (AI) and Machine Learning (ML) is shifting hydrogel development from empirical iteration toward predictive engineering, effectively addressing the “trial-and-error” bottleneck in complex optimization. Recent advances in generative design have produced high-throughput platforms such as “AMP-hydrogel-Designer” to identify bioactive sequences [199]. To enhance predictive precision, dedicated algorithmic frameworks—such as Gaussian process regression and artificial neural networks (ANNs) [200]—are now utilized to model the non-linear relationships between hydrogel composition and storage modulus (G’). For instance, by leveraging specialized hybrid AI platforms like FormRules version 4.03, researchers can identify the specific polymer concentrations required to maximize mucosal adhesion and injectability for targeted delivery [201, 202].

To bridge the translational gap, we propose an integrated validation workflow that commences with the in silico optimization of monomer-bioactive coupling via generative models [200], which subsequently informs an automated physical screening phase. This characterization stage leverages physics-guided ML and automated sensing to achieve a 70-fold increase in rheological testing throughput (approximately 24 s/sample) [203]. The process then transitions to biological simulation, utilizing patient-derived organoids (PDOs) within complex stromal-immune co-cultures to evaluate the modulation of the “viscoelastic niche“ [204]. This systematic pipeline culminates in preclinical validation using orthotopic or PDX models to confirm both locoregional therapeutic synergy and systemic immunity [205].

Complementing these predictions, PDOs have become indispensable for validating AI-driven architectures. Unlike reductionist 2D cultures, hydrogel-based 3D models more faithfully recapitulate mechanotransductive signaling—particularly the YAP1/ABCB1 loop—linked to stiffness-dependent drug resistance in breast and pancreatic malignancies [206–208]. A compelling example of this efficiency is the use of miniaturized experimental tactics combined with ML to optimize photodegradable hydrogels; researchers successfully optimized over 13,440 hydrogel combinations using only 13 iterative experiments [209]. Furthermore, the development of the AI-HTPCSS (AI-assisted high-throughput printing-condition-screening system) enables the rapid identification of optimal formulations that maintain cell viability while achieving consistent shape fidelity in 3D-printed scaffolds [205, 210]. In this setting, the AI–organoid nexus supports high-throughput screening of “resistance-reversal first, precision-killing later” strategies, connecting benchtop design with patient-tailored precision oncology [211, 212].

## Conclusion

In summary, the clinical translation of next-generation hydrogels depends on a shift from material-centric innovation to a clinically-oriented development paradigm. By integrating advanced biomanufacturing, data-guided predictive design, and patient-derived organoid validation, these platforms are engineered to address the physical, metabolic, and cellular barriers that sustain multidrug resistance. The primary objective remains clinical viability, which requires manufacturing standardization, rigorous validation in realistic models, and designs that demonstrate sustained efficacy *in vivo.* Together, these advances provide a feasible framework to bridge the gap between laboratory research and clinical application, advancing toward patient-specific precision oncology.

## Supplementary Information

Supplementary Material 1.

## Data Availability

No datasets were generated or analysed during the current study.

## Abbreviations

ABC: ATP-binding cassette
ACT: Adoptive cell therapy
AI: Artificial Intelligence
CAR: Chimeric antigen receptor
CD40L: CD40 ligand
CDT: Chemodynamic therapy
CMC: Chemistry, Manufacturing, and Controls
CQAs: Critical quality attributes
CRISPR–Cas9: clustered regularly interspaced short palindromic repeats–Cas9
CRS: Cytokine release syndrome
cDC1: Type 1 conventional dendritic cells
cGAS: Cyclic GMP–AMP synthase
DC: Dendritic cell(s)
DCA: Dichloroacetate
ECM: Extracellular matrix
EGFR: Epidermal growth factor receptor
EMT: Epithelial–mesenchymal transition
EPR: Enhanced permeability and retention
FBR: Foreign body response
FLT3L: Fms-like tyrosine kinase 3 ligand
GBM: Glioblastoma
GMP: Good Manufacturing Practice
GOx: Glucose oxidase
G′: Storage modulus
HA: Hyaluronic acid
Hb: Hemoglobin
HBOCs: Hemoglobin-based oxygen carriers
HCC: Hepatocellular carcinoma
HIF-1α: Hypoxia-inducible factor-1α
HIFs: Hypoxia-Inducible Factors
ICB: Immune checkpoint blockade
IDO: Indoleamine 2,3-dioxygenase
lncRNAs: Long noncoding RNAs
mRNA: Messenger RNA
MDR: Multidrug resistance
MDSCs: Myeloid-derived suppressor cells
ML: Machine Learning
MOFs: Metal–organic frameworks
NIR: Near-infrared
NO: Nitric oxide
NTPA: Nucleus-targeting amphiphilic peptides
OXPHOS: Oxidative phosphorylation
P50: Oxygen partial pressure at 50% hemoglobin saturation
PDT: Photodynamic therapy
PFCs: Perfluorocarbons
P-gp: P-glycoprotein
PDOs: Patient-derived organoids
PDAC: Pancreatic ductal adenocarcinoma
PI3K-γ: Phosphoinositide 3-kinase gamma
PLNPs: Persistent luminescence nanoparticles
QbD: Quality by Design
QTPP: Quality Target Product Profile
ROS: Reactive oxygen species
STING: Stimulator of interferon genes
TAZ: Transcriptional coactivator with PDZ-binding motif
TAMs: Tumor-associated macrophages
TEM: Effector memory T cells
TdLN: Tumor-draining lymph nodes
TILs: Tumor-infiltrating lymphocytes
TME: Tumor microenvironment
USP9X: Ubiquitin-specific peptidase 9X
YAP: Yes-associated protein
YAP1: Yes-associated protein 1
ΔΨm: Mitochondrial membrane potential
